# Loss of periostin occurs in aging adipose tissue of mice and its genetic ablation impairs adipose tissue lipid metabolism

**DOI:** 10.1111/acel.12810

**Published:** 2018-08-07

**Authors:** Antonia Graja, Francisco Garcia‐Carrizo, Anne‐Marie Jank, Sabrina Gohlke, Thomas H. Ambrosi, Wenke Jonas, Siegfried Ussar, Matthias Kern, Annette Schürmann, Krasimira Aleksandrova, Matthias Blüher, Tim J. Schulz

**Affiliations:** ^1^ Department of Adipocyte Development and Nutrition German Institute of Human Nutrition Potsdam‐Rehbrücke Germany; ^2^ University of Potsdam, Institute of Nutritional Science Potsdam‐Rehbrücke Germany; ^3^ Department of Experimental Diabetology German Institute of Human Nutrition Potsdam‐Rehbrücke Germany; ^4^ German Center for Diabetes Research (DZD) Munich‐Neuherberg Germany; ^5^ JRG Adipocytes and Metabolism Institute for Diabetes and Obesity Helmholtz Center Munich Garching Germany; ^6^ Department of Medicine University of Leipzig Leipzig Germany; ^7^ Nutrition, Immunity and Metabolism Senior Scientist Group German Institute of Human Nutrition Potsdam‐Rehbrücke Germany

**Keywords:** adipogenic progenitor cells, adipose tissue, aging, extracellular matrix, fatty acid metabolism, periostin

## Abstract

Remodeling of the extracellular matrix is a key component of the metabolic adaptations of adipose tissue in response to dietary and physiological challenges. Disruption of its integrity is a well‐known aspect of adipose tissue dysfunction, for instance, during aging and obesity. Adipocyte regeneration from a tissue‐resident pool of mesenchymal stem cells is part of normal tissue homeostasis. Among the pathophysiological consequences of adipogenic stem cell aging, characteristic changes in the secretory phenotype, which includes matrix‐modifying proteins, have been described. Here, we show that the expression of the matricellular protein periostin, a component of the extracellular matrix produced and secreted by adipose tissue‐resident interstitial cells, is markedly decreased in aged brown and white adipose tissue depots. Using a mouse model, we demonstrate that the adaptation of adipose tissue to adrenergic stimulation and high‐fat diet feeding is impaired in animals with systemic ablation of the gene encoding for periostin. Our data suggest that loss of periostin attenuates lipid metabolism in adipose tissue, thus recapitulating one aspect of age‐related metabolic dysfunction. In human white adipose tissue, periostin expression showed an unexpected positive correlation with age of study participants. This correlation, however, was no longer evident after adjusting for BMI or plasma lipid and liver function biomarkers. These findings taken together suggest that age‐related alterations of the adipose tissue extracellular matrix may contribute to the development of metabolic disease by negatively affecting nutrient homeostasis.

## 1 INTRODUCTION

Aging is associated with increased body weight gain (Chumlea et al., 2002). Overweight is a risk factor for the metabolic syndrome and promotes progression of different pathologies (Rosen & Spiegelman, 2014). White adipose tissue (WAT) stores energy as triglycerides, whereas brown adipose tissue (BAT) dissipates energy in the form of heat, and may thus protect against hypothermia. The extracellular matrix (ECM) provides a structural scaffold for adipose depots. It functions as a gatekeeper that regulates the exchange of adipokines and growth factors between fat cells and the circulation (Lu, Takai, Weaver, & Werb, 2011; Mariman & Wang, 2010). Aside from adipocytes, adipose tissue‐resident mesenchymal stem cells are a main source of secreted signals. Their secretory profile, which includes matrix‐modifying enzymes, changes significantly with increased age (Tchkonia et al., 2010). ECM remodeling during adipose tissue expansion is essential to allow sufficient de novo vascularization and control of adipocyte size (Lin & Kang, 2016). For instance, loss of collagen 6 increases expansion of adipocytes and improves metabolic features suggesting that excessive matrix accumulation is a pathological feature of obesity (Khan et al., 2009). The 90 kDa matricellular protein periostin is encoded by the *Postn* gene. It is secreted into the matrix space and can activate integrins (Itg), specifically heterodimers of Itgαv with Itgβ1, Itgβ3, or Itgβ5, which in turn regulate processes such as proliferation and differentiation (Idolazzi et al., 2017). Additionally, periostin interacts with structural collagens, thereby influencing mechanical properties of the ECM (Gillan et al., 2002; Kudo, 2011; Norris et al., 2007). Its expression is controlled by mechanical stimuli and growth factors, such as transforming growth factor beta, bone morphogenetic protein 2, among other signaling molecules (Aukkarasongsup, Haruyama, Matsumoto, Shiga, & Moriyama, 2013; Ji et al., 2000).

Here, we investigated the role of periostin in BAT and WAT function and its potential involvement in lipid metabolism during aging. While aging results in a marked downregulation of *Postn* expression, metabolic interventions that require adipose tissue remodeling induce *Postn* gene expression. In line with these observations, systemic deletion of *Postn* in mice resulted in loss of adipose tissue (AT) mass after cold exposure, reduced AT expansion and adipocyte size during high‐fat diet (HFD) feeding, and impaired lipolytic enzyme activity after acute β3‐adrenergic stimulation. Unexpectedly, expression of periostin mRNA in human WAT was positively associated with aging but inversely correlated with BMI. The age‐related correlation could partially be explained by changes to BMI, as well as plasma lipid and liver biomarkers.

## 2 RESULTS

### 2.1 Aging and metabolic interventions regulate the expression of periostin in adipose tissue

**Figure 1:**
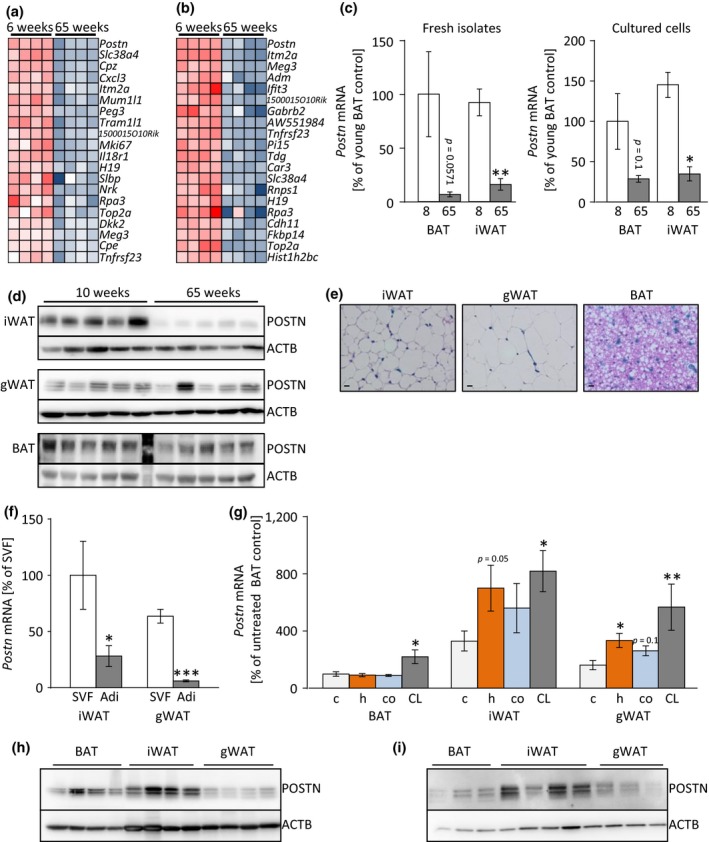
The matricellular protein periostin is regulated by aging and metabolic challenges. (a, b) Heatmap of top 20 downregulated genes expressed in BAT‐ (a) and iWAT‐derived (b) APCs comparing old (65 weeks) to young (6 weeks) murine samples. (c) Periostin gene expression in BAT‐ and iWAT‐derived, FACS‐purified APCs of young (8 weeks, white bars) and old (65 weeks, gray bars) mice directly after isolation (left panel) and after *in vitro* cultivation (right panel; *n* = 3–6). (d) Western blot analysis of POSTN and β‐actin (ACTB) expression in iWAT (top panels), gWAT (middle panels), and BAT (lower panels) of young (8 weeks) compared to old (65 weeks) mice (*n* = 5). A protein marker lane is visible between the young and old tissue samples in the depiction of the BAT samples shown here. (e) Representative X‐Gal/H&E staining (400× magnification; Scale bar: 10 μm) in periostin‐driven LacZ reporter mouse strain in iWAT, gWAT, and BAT. (f) *Postn* mRNA in freshly isolated stromal‐vascular‐fraction (SVF, white bars) compared to isolated adipocytes (Adi, gray bars) purified from iWAT and gWAT of young C57BL/6 J mice (*n* = 8). (g) *Postn* mRNA in BAT, iWAT, and gWAT in young control mice (c; light gray bars), after 6 weeks of HFD (h; orange bars), after cold exposure (co; blue bars), or 180 min after injection of the β3‐adrenergic receptor agonist, CL316,243 (CL; dark gray bars; *n* = 7–9). (h, i) Western blot analysis of POSTN protein expression normalized to β‐actin (ACTB) in BAT, iWAT, and gWAT of male (h) and female (i) C57Bl/6 J wild‐type mice. Mean ± *SEM*; *p < 0.05, **p < 0.01, ***p < 0.001, assessed by unpaired *t* test or Mann–Whitney *U* test

To test whether aging negatively affects the function of adipose tissue‐resident stem/progenitor cells, we isolated adipogenic progenitors cells (APCs) from BAT and inguinal WAT (iWAT) by flow cytometry for microarray‐based analysis (Schulz et al., 2011). Aside from a number of genes belonging to an imprinted gene network that has been linked to adiposity (Morita et al., 2016), we found the gene encoding for periostin (*Postn*) to be the most strongly downregulated candidate gene (Figure 1a,b). The age‐related reduction in *Postn* mRNA could be verified by real‐time quantitative PCR (Figure 1c). Analysis of periostin protein levels confirmed the negative effect of aging in iWAT and BAT, but not in gonadal WAT (gWAT; Figure 1d, Supporting Information Figure S1a–c). To determine the distribution of periostin expression in adipose tissues, a transgenic mouse strain with β‐galactosidase (lacZ) knock‐in was used for histological analysis. The reporter is expressed from the *Postn* gene locus in lieu of the native protein. These analyses revealed a marked reporter activity in cells adherent to blood vessels and in interstitial cells (Figure 1e). The predominant expression in nonadipocytes was confirmed by increased *Postn* levels in the stromal‐vascular fraction (SVF) compared to mature adipocytes in iWAT and gWAT (Figure 1f). To test whether *Postn* expression was regulated by physiological stimuli in AT, mRNA levels were assessed after 6 weeks of HFD, short‐term cold exposure, and following acute beta‐adrenergic stimulation with the β3‐adrenergic receptor agonist, CL316,243 (CL). HFD and CL injections resulted in upregulation of *Postn* in iWAT and gWAT, and a similar trend was found after cold, while only beta‐adrenergic stimulation increased *Postn* expression in BAT (Figure 1g). Human periostin protein was found to be higher in iWAT and BAT compared to gWAT in males and but comparable between depots in female mice (Figure 1h,i, Supporting Information Figure S1d,e). In summary, these findings suggest that periostin may be involved in AT remodeling following physiological and dietary cues, whereas this effect is attenuated with increased age.

### 2.2 Genetic ablation of *Postn* results in reduced body size but normal AT distribution

**Figure 2:**
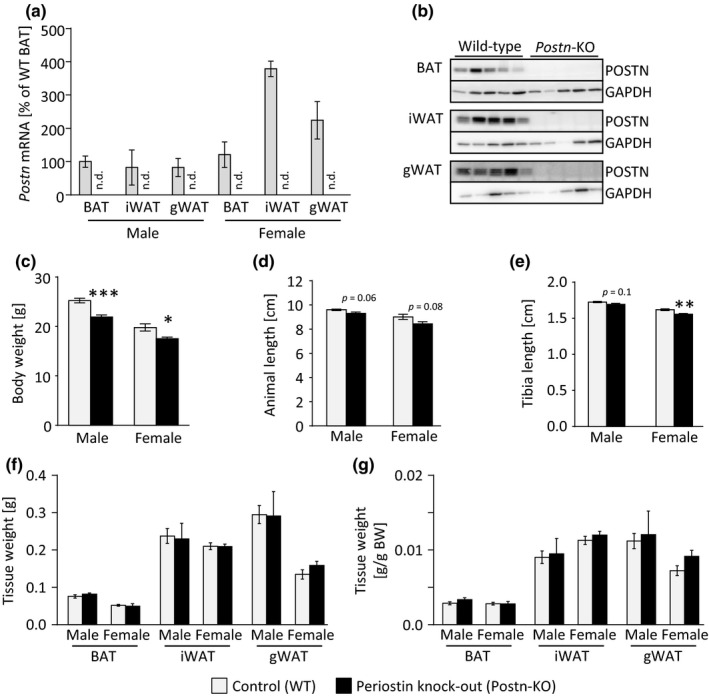
Genetic deletion of *Postn* leads to mild growth retardation but does not affect AT development. (a) *Postn* gene expression in BAT, iWAT, and gWAT of male mice comparing wild‐type (WT, gray bars) and *Postn*‐KO animals (*n* = 3–4; n.d.—not detectable). (b) Protein expression of POSTN in different AT depots, for example, BAT, iWAT, gWAT, of male WT and *Postn*‐KO animals normalized to glyceraldehyde 3‐phosphate dehydrogenase (GAPDH) expression. (c) Body weight of male and female mice comparing WT and *Postn*‐KO mice (*n* = 8–12). Gray bars represent WT controls, black bars represent *Postn*‐KO mice, applies to all subsequent panels. (d) Animal length of male and female WT and *Postn*‐KO animals (*n* = 4–5). (e) Tibia lengths of male and female WT and *Postn*‐KO animals (*n* = 4–7). (f, g) AT depot weights of male and female WT and *Postn*‐KO animals prior to normalization (f) or normalized to body weight (g; *n* = 3–6). Mean ± *SEM*; *p < 0.05, **p < 0.01, ***p < 0.001, assessed by unpaired *t* test

To directly address the potential role of periostin in AT biology, we generated homozygous LacZ reporter mice, thereby creating a strain with whole‐body deletion of *Postn* gene expression (Figure 2a,b). Recapitulating previous observations (Oshima et al., 2002; Rios et al., 2005), *Postn*‐KO mice displayed significantly reduced body weight as well as body and tibia lengths (Figure 2c–e). AT depots displayed no size differences implying that a reduction in periostin does not affect AT development (Figure 2f,g). Brown and white fat depots displayed normal morphology and ECM distribution, and unchanged gene expression patterns of ECM components, white and brown adipogenesis, or senescence (Supporting Information Figure S2). Similarly, pre‐adipocytes isolated from iWAT and BAT of knockout mice and wild‐type littermates displayed normal differentiation and adipogenic gene expression patterns suggesting intact adipogenic differentiation capacities (Supporting Information Figure S3).

### 2.3 Impaired cold tolerance and loss of WAT mass in *Postn*‐KO mice during cold exposure

**Figure 3:**
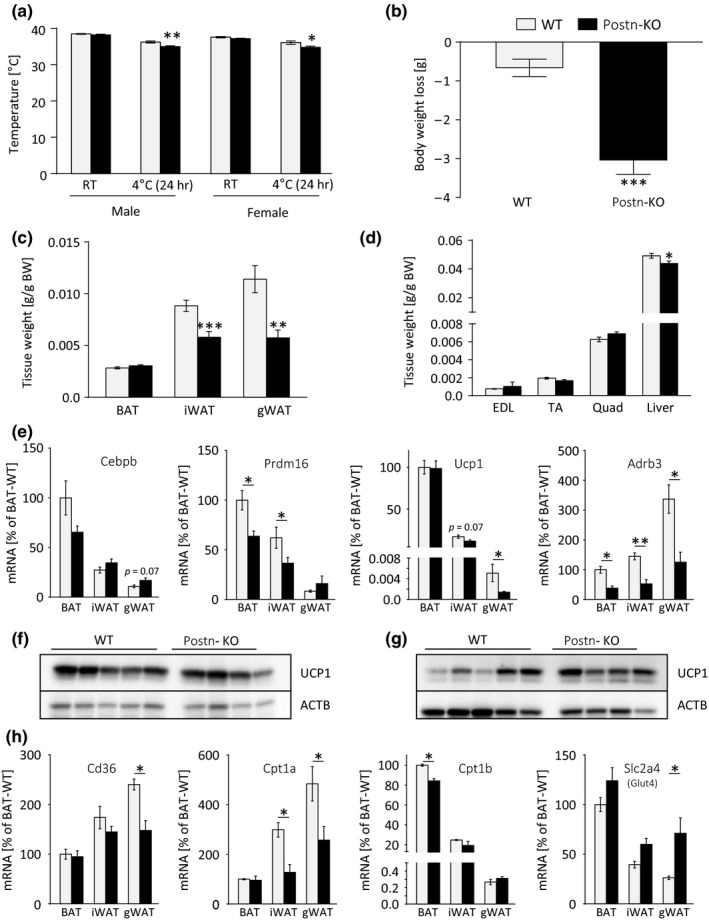
Mild impairment of cold‐induced thermogenesis in periostin‐deficient mice. (a) Rectal temperatures of male and female WT and *Postn*‐KO animals at room temperature (RT) and after 24 hr cold exposure (*n* = 5–12). Gray bars represent WT controls, black bars represent *Postn*‐KO mice, applies to all subsequent panels. (b) Body weight loss of male WT and *Postn‐*deleted mice after 72 hr of cold exposure (*n* = 10–11). (c) BAT, iWAT, and gWAT depot weights normalized to body weight of male WT and *Postn*‐KO mice after cold exposure for 72 hr (*n* = 10–11). (d) Body weight‐normalized tissue weights of extensor digitorum longus (EDL), tibialis anterior (TA), and quadriceps femoris (Quad) muscles and liver in male WT and *Postn*‐KO animals after cold exposure for 72 hr (*n* = 10–11). (e) Gene expression analysis of the brown/beige adipogenesis marker genes *Cebpb*,* Prdm16*,* Ucp1,* and *Adrb3* in BAT, iWAT, and gWAT of male *Postn*‐deficient mice after cold exposure (*n* = 5–7). (f, g) Western blot analysis of UCP1 in BAT (f) and iWAT (g) of male WT and *Postn*‐KO animals after 72 hr cold exposure normalized to β‐actin (ACTB). (h) Gene expression analysis *Cd36*,* Cpt1a*,* Cpt1b,* and *Scl2a4 (Glut4)* in BAT, iWAT, and gWAT of male *Postn*‐deficient mice after cold exposure (*n* = 5–7). Data are shown as mean ± *SEM*. *p < 0.05, **p < 0.01, ***p < 0.001 compared with WT animals assessed by unpaired *t* test or Mann–Whitney *U* test

Cold exposure leads to substantial remodeling of WAT and, to some extent, BAT. A significant decrease in thermogenesis in BAT and browning of WAT has been described as a result of increased age in humans and rodents (Florez‐Duquet, Horwitz, & McDonald, 1998; Pfannenberg et al., 2010; Yoneshiro et al., 2011). We therefore examined the effects of cold exposure in *Postn*‐knockout mice and observed a rapid loss of body weight within 72 hr of cold exposure which prevented further long‐term treatments (Figure 3a,b). Further analysis revealed that the weight loss during cold exposure was due to a significant reduction in WAT mass, but also liver mass, rather than weight loss in other tissues (Figure 3c,d). Plasma lipid analysis revealed a marked decrease in circulating lipid levels during cold but no consistent genotype‐dependent changes were observed in either male or female knockout mice (Supporting Information Figure S4a). These data suggest that systemic lipid homeostasis was regulated in a gender‐specific manner in knockout animals but was unlikely to contribute to the observed phenotype. As brown adipocyte nonshivering thermogenesis controls body temperature, we assessed expression of the brown fat‐defining marker uncoupling protein 1 (Ucp1) but found it to be unchanged on mRNA or protein levels in BAT (Figure 3e,f). In iWAT, *Ucp1* mRNA and protein levels displayed no significant changes while significantly reduced, but overall very low, expression of *Ucp1* mRNA in gWAT was observed (Figure 3e,g, Supporting Information Figure S4b,c). These unexpected results rule out reduced UCP1 expression as a cause of hypothermia in cold‐exposed *Postn*‐KO mice. Expression of CCAAT/enhancer‐binding protein beta (*Cebpb*) and PR domain containing 16 *(Prdm16*) only trended toward downregulation after cold exposure (Figure 3e). However, expression of β3‐adrenergic receptor (*Adrb3*) was significantly reduced in BAT and WAT of cold‐exposed *Postn*‐KO mice. Additionally, we measured reduced expression of lipid metabolism genes such as the lipid transporter *Cd36* in gWAT, and the respective main isoforms of carnitine‐palmitoyltransferase 1, *Cpt1a* in gWAT and iWAT and *Cpt1b* in BAT. At the same time, expression of glucose transporter 4 (*Glut4*;* Slc2a4*) was induced in WAT and could potentially explain the reduced glucose levels observed in cold‐exposed knockouts compared to wild‐type littermates (Figure 3h, Supporting Information Figure S4d). These data taken together suggest a potential but limited defect of brown/beige adipocyte function that may contribute the lower thermogenic potential and cold tolerance of *Postn*‐KO mice. Moreover, attenuated lipid mobilization and metabolism may contribute to this defect.

### 2.4 Deletion of *Postn* leads to impaired HSL activation in AT during adrenergic stimulation

**Figure 4:**
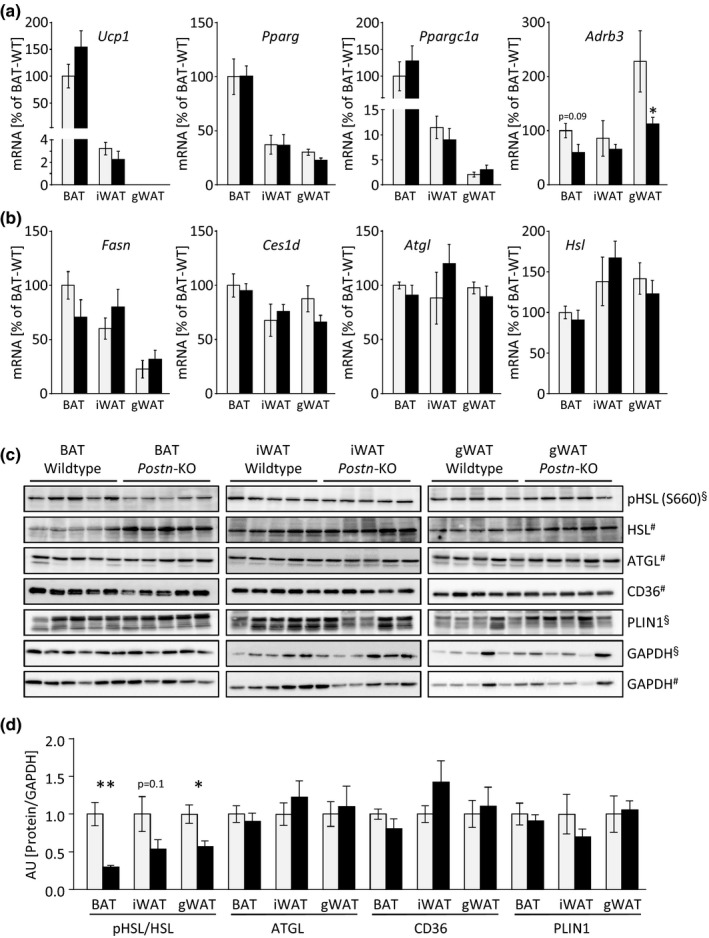
Deletion of *Postn* leads to an impaired lipid metabolism after acute β‐adrenergic stimulation. (a, b) Gene expression of genes associated with brown adipogenesis, *Ucp1, Pparg, Ppargc1a, Adrb3* (a) and lipid metabolism, *Fasn*,* Ces1d*,* Atgl,* and *Hsl* (b) in BAT, iWAT, and gWAT of male WT and *Postn*‐KO animals 180 min after i.p.‐injection of β3‐adrenergic receptor agonist, CL316,243. Gray bars indicate control mice, black bars indicate *Postn*‐KO mice, applies to all panels. (c, d) Western blot analysis and quantification of proteins associated with lipid metabolism, pHSL, HSL, ATGL, CD36, and PLIN1 in BAT, iWAT, and gWAT. Western blot signals were normalized to glyceraldehyde 3‐phosphate dehydrogenase (GAPDH). ^§^pHSL and Plin1 antibodies were probed on the same membrane and normalized to GAPDH in the upper panel; ^#^HSL, ATGL, and CD36 were probed on a separate membrane and normalized to GAPDH in the lower panel. Data are shown as mean ± *SEM*. *n* = 5. *p < 0.05, **p < 0.01 compared with WT animals assessed by Mann–Whitney *U* test

To further address the hypothesis of dysfunctional lipid metabolism in *Postn*‐depleted AT, lipolysis in male *Postn*‐KO mice and WT littermates was induced by injections of the β3‐adrenergic receptor agonist, CL316,243 (CL). During this acute stimulation, the deletion of *Postn* did not affect expression of brown and general adipogenic genes, such as *Ucp1*, peroxisome proliferator‐activated receptor gamma (*Pparg*), or peroxisome proliferator‐activated receptor gamma coactivator 1‐alpha (*Pgc1a*), or plasma lipids (Figure 4a, Supporting Information Figure S5a). However, *Postn*‐KO mice exhibited a mild decrease in *Adrb3* expression which was consistent with the observation in cold‐exposed AT samples (Figure 3e and 4a). Genes of the lipogenic or lipolytic pathways, such as fatty acid synthase (*Fasn*), triacylglycerol hydrolase (*Ces1d*), adipose tissue triglyceride lipase (*Atgl*), and hormone‐sensitive lipase (*Hsl*), were also found to be unchanged on mRNA level (Figure 4b). As these lipolytic genes are also regulated post‐transcriptionally, we next analyzed protein levels of phosphorylated and basal HSL, ATGL, the lipid transporter CD36, and the lipid droplet coating protein perilipin‐1 (PLIN1). While neither ATGL, nor CD36, nor PLIN1 was changed, total HSL protein was significantly increased in BAT of KO mice. In contrast to this, the levels of phosphorylated, e.g. activated HSL (pHSL) were significantly decreased in BAT of *Postn*‐KO mice, and altogether showed a reduced pHSL/HSL ratio (Figure 4c,d, Supporting Information Figure S5b). Similar effects, albeit at a lower magnitude, were also observed in iWAT and gWAT (Figure 4c,d, Supporting Information Figure S5b). These data taken together further support the conclusion that lipid mobilization may be defective in AT of *Postn*‐KO mice.

### 2.5 Loss of periostin inhibits AT expansion during high‐fat diet feeding

**Figure 5:**
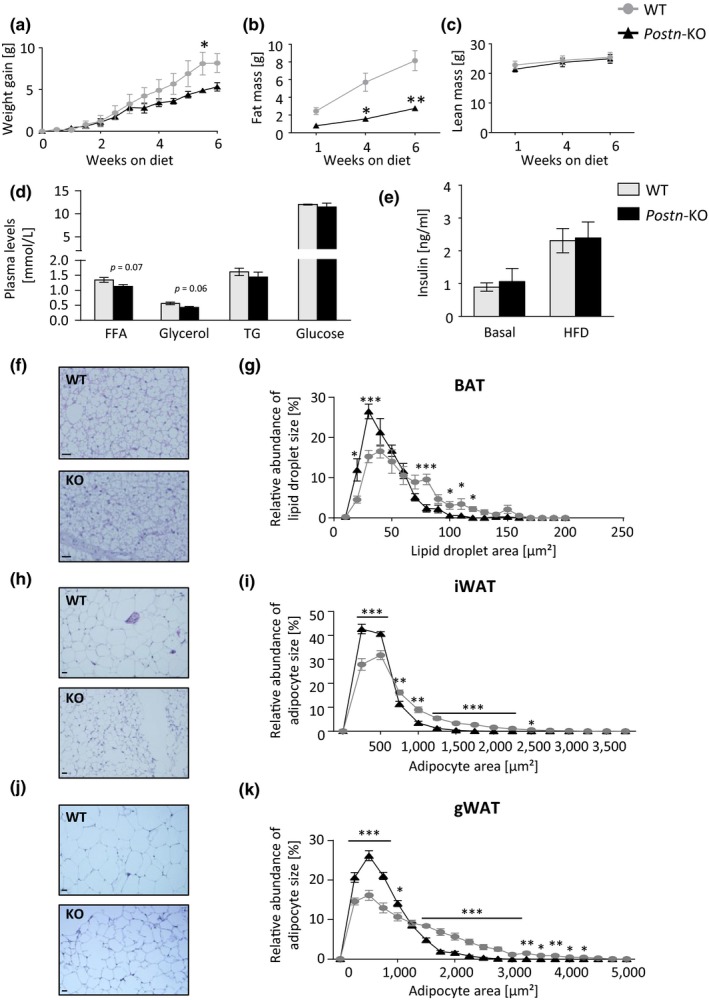
*Postn* deficiency impairs adipose tissue expansion during high‐fat diet feeding. (a) Body weight gain of male WT and *Postn*‐KO animals during 6 weeks of HFD feeding. Gray lines/circles/bars represent WT controls, black lines/triangles/bars represent *Postn*‐KO mice, applies to all subsequent panels. (b, c) NMR analysis of male WT and *Postn*‐KO animals assessing total fat mass (b) and lean mass (c). (d) Plasma levels of FFA, glycerol, TG, and glucose in male WT and *Postn*‐depleted animals after 6 weeks of HFD. (e) Plasma levels of insulin with and without high‐fat diet feeding in male WT and *Postn*‐depleted animals. (f) Representative images of H&E staining (200× magnification; scale bar: 20 μm, applies to all subsequent images of BAT) of BAT of male WT (upper panel) and male *Postn*‐KO mice (lower panel). (g) Quantitative analysis of lipid droplet size in BAT sections after H&E staining of male WT and knockout animals after 6 weeks of HFD as shown in previous panel. (h) Representative H&E staining (100× magnification; scale bar: 20 μm, applies to all subsequent images of WAT) of iWAT of male WT (upper panel) and male *Postn*‐KO mice (lower panel). (i) Quantitative analysis of adipocyte size analysis of male iWAT comparing WT to *Postn*‐KO animals after 6 weeks of HFD from images as shown in previous panel. (j) Representative H&E staining (100× magnification) of gWAT of male WT (upper panel) and male *Postn*‐KO mice (lower panel). (k) Adipocyte size analysis of male gWAT comparing WT to *Postn*‐KO animals after 6 weeks of HFD from images as shown in previous panel. Data are shown as mean ± *SEM* (*n* = 5–7). *p < 0.05, **p < 0.01, ***p < 0.001 as assessed by two‐way ANOVA with Bonferroni *post hoc* test (a–c, g, i, k) and unpaired *t* test (d, e)

To elucidate the effects of *Postn* deletion on other aspects of lipid metabolism, male and female animals were fed a HFD for 6 weeks, starting at an age of 8 weeks. Interestingly, male *Postn*‐KO mice gained significantly less weight compared to WT control mice although this effect was not observed in female mice (Figure 5a, Supporting Information Figure S6a). Both genders displayed a marked attenuation of fat mass gain during six weeks of HFD feeding. No differences in lean mass were found in male knockout mice. In females, lean mass was reduced throughout the course but the magnitude of the reduction was not affected (Figure 5b,c, Supporting Information Figures S6b,c and S7a,b). Food intakes in mice fed control or HFD were unchanged after normalization to body weight and basal energy expenditure was also not changed in animals on control diet (Supporting Information Figure S7c–e). Plasma free fatty acid (FFA) and glycerol levels showed a trend towards a reduction in male *Postn*‐KO mice, suggesting that alterations to lipid metabolism may be involved in this attenuation. However, no differences were found in female mice (Figure 5d, Supporting Information Figure S6d). Insulin levels were unchanged in either sex on control diet or after HFD (Figure 5e, Supporting Information Figure S6e). Interestingly, histological analysis of BAT revealed significantly smaller lipid droplet sizes and significantly reduced adipocyte sizes in iWAT and gWAT after 6 weeks of HFD in male and, to a lesser extent, in female knockout mice (Figure 5f–k, Supporting Information Figure S6f–K). To test whether the HFD‐related loss of fat mass could be explained by changes to thermogenesis or ECM function, gene expression was assessed but found to be unchanged in either BAT or WAT depots (Supporting Information Figure S8). Together, these data suggest that the deletion of periostin leads to impaired lipid metabolism in adipose tissues during HFD feeding which is consistent with the defective metabolic response observed during cold exposure.

### 2.6 Periostin expression in human white adipose tissue is regulated by aging and obesity

**Figure 6:**
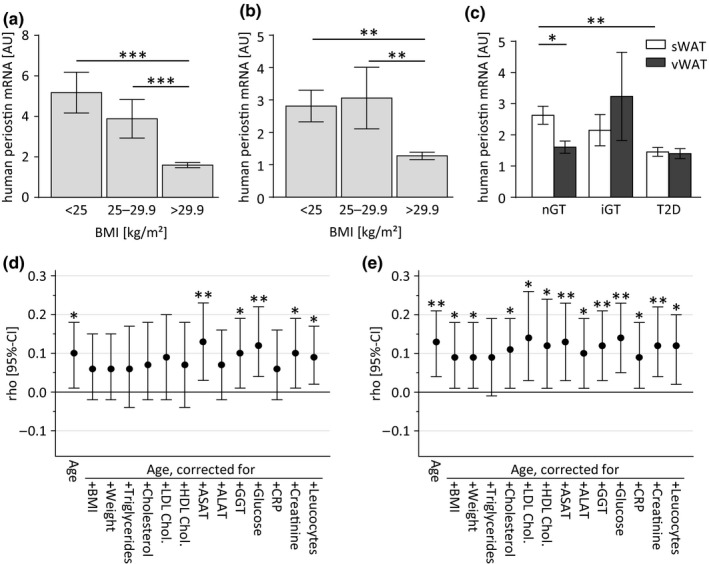
Human periostin mRNA expression in different fat depots is negatively associated with obesity and parameters of glucose metabolism in humans. (a, b) Gene expression analysis of periostin mRNA in human sWAT (a) and human vWAT (b) biopsies collected from normal weight (body mass index (BMI) <25 kg/m^2^), overweight (BMI 25–29.9 kg/m^2^), and obese (BMI > 29.9 kg/m^2^) subjects. (c) Human periostin mRNA levels in sWAT (white bars) and vWAT (gray bars) of humans with normal glucose tolerance, impaired glucose tolerance and type 2 diabetes. Data are shown as mean ± *SEM*. *n* = 12–408. *p < 0.05, **p < 0.01, ***p < 0.001 assessed by one‐way (a, b) or two‐way (c) ANOVA with Bonferroni *post hoc* test. (d, e) Graphical depictions of Spearman partial correlation coefficients (*ρ*) and 95% confidence intervals (CI) of associations between participant age and human periostin mRNA expression in sWAT (panel d) and vWAT (panel e) following adjustment for body mass index (BMI), or lipid and liver function parameters (data shown in Table 1). *p < 0.05, **p < 0.01 indicate significant correlations

To determine a potential relationship of periostin expression and AT lipid homeostasis in humans, we analyzed the human periostin mRNA expression in WAT biopsies (*n* = 471) of lean (BMI < 25 kg/m^2^), overweight (BMI 25–29.9 kg/m^2^), and obese subjects (BMI > 29.9 kg/m^2^). A significant inverse correlation between human periostin mRNA levels and obesity was observed in subcutaneous WAT (sWAT) as well as in visceral WAT (vWAT; Figure 6a,b). In healthy subjects with normal glucose tolerance, expression of human periostin is significantly higher in sWAT compared to vWAT, and this difference was blunted in patients with impaired glucose tolerance or overt type 2 diabetes (Figure 6c). In contrast to murine AT, a positive correlation between participant age and human periostin mRNA was found in both human AT depots (Table 1, Figure 6d,e). However, this correlation was no longer statistically significant following adjustment by BMI in sWAT and showed reduced significance level in vWAT, suggesting that this unexpected observation may be partially explained by participants’ adiposity status (Table 1, Figure 6d,e). Moreover, correction of triglycerides removed the age‐related correlation in both WAT depots, suggesting an interaction of lipid metabolism and aging. Further analysis following stratification according to BMI categories revealed that the positive correlation between participants’ age and human periostin mRNA was present only in overweight individuals (25 < BMI kg/m^2^≤ 30), while an inverse correlation was observed in normal weight individuals (BMI < 25 g/m^2^), although it should be noted that these differences were not significant due to the comparably low sample size in the normal weight and overweight groups (Supporting Information Table S1). In addition to BMI, the correlations with age were further explained by plasma lipids and, to a more limited extent, also by liver parameters (i.e., ASAT, ALAT; Table 1). Collectively, these data suggest that expression of human periostin mRNA in human WAT interacts with age and age‐associated phenotypes depending on individual levels of adiposity and metabolic health.

**Table 1:** Spearman partial correlation coefficients (*ρ*) and 95% confidence intervals (CI) to depict associations between participant age and human periostin mRNA expression in sWAT and vWAT following adjustment for body mass index (BMI), or lipid and liver function parameters

Variable	human periostin mRNA expression in human sWAT		human periostin mRNA expression in human vWAT	
	*ρ* (95% CI)	*p*‐Value	*ρ* (95% CI)	*p*‐Value
Age (n = 471)	0.10 (0.1; 0.18)	0.03	0.13 (0.04; 0.21)	0.005
Age (adj. for BMI; n = 471)	0.06 (−0.02; 0.15)	0.14*	0.09 (0.01; 0.18)	0.03
Age (adj. for body weight; n = 469)	0.06 (−0.02; 0.15)	0.17*	0.09 (0.01; 0.18)	0.04
Age (adj. for triglycerides; n = 342)	0.06 (−0.04; 0.17)	0.20*	0.09 (−0.01;0.19)	0.08*
Age (adj. for total cholesterol; n = 338)	0.07 (−0.02; 0.18)	0.15*	0.11 (0.01; 0.19)	0.03
Age (adj. for LDL cholesterol; n = 282)	0.09 (−0.02; 0.20)	0.12*	0.14 (0.03; 0.26)	0.01
Age (adj. for HDL cholesterol; n = 281)	0.07 (−0.04; 0.18)	0.24*	0.12 (0.01; 0.24)	0.03
Age (adj. for ASAT; n = 395)	0.13 (0.03; 0.23)	0.008	0.13 (0.03; 0.23)	0.007
Age (adj. for ALAT; n = 461)	0.07 (−0.02; 0.16)	0.10*	0.10 (0.01;0.19)	0.02
Age (adj. for GGT; n = 456)	0.10 (0.01; 0.19)	0.02	0.12 (0.03; 0.21)	0.008
Age (adj. for fasting glucose; n = 439)	0.12 (0.04; 0.22)	0.007	0.14 (0.05; 0.23)	0.002
Age (adj. for CRP; n = 461)	0.06 (−0.02;0.16)	0.16*	0.09 (0.01; 0.18)	0.04
Age (adj. for creatinine; n = 462)	0.10 (0.01; 0.19)	0.02	0.12 (0.04; 0.22)	0.005
Age (adj. for leukocytes; n = 466)	0.09 (0.02; 0.17)	0.05	0.12 (0.02; 0.20)	0.01

*Adjustment for biomarker leads to loss of statistical significance.

## 3 DISCUSSION

Aging is associated with defective ECM function leading to tissue fibrosis, that is, an excessive accumulation of fibrous connective tissue and ECM components (Wynn, 2007). Similar alterations have also been described in adipose tissue (Sun, Tordjman, Clement, & Scherer, 2013). Interestingly, undifferentiated adipogenic progenitor cells are a major source of ECM components in AT (Kubo, Kaidzu, Nakajima, Takenouchi, & Nakamura, 2000; Nakajima, Yamaguchi, Ozutsumi, & Aso, 1998), suggesting that alterations in progenitor cell function with increased age may contribute to homeostatic changes in the tissue matrix (Tchkonia et al., 2010). Periostin is a secreted factor and its binding integrin heterodimers has been shown to activate protein kinase B and the focal adhesion kinase‐mediated intracellular signaling (Idolazzi et al., 2017; Kuhn et al., 2007; Morra & Moch, 2011).

As aging resulted in a significant downregulation of periostin expression in AT, we decided to analyze BAT and WAT in a mouse model with genetic ablation of *Postn*. This deletion resulted in decreased body size but did not impair the development of BAT or WAT depots. Our data are thus consistent with previous reports on the role of periostin in bone formation (Rios et al., 2005). While adipose tissue appeared normal under basal conditions, short‐term cold exposure revealed a defective capacity for thermoregulation. The specific loss of adipose tissue mass after cold was accompanied by only minor alterations in the plasma lipid profiles. While basal energy expenditure was unchanged, we cannot rule out that cold exposure or HFD may introduce alterations of this parameter that could contribute to loss of fat mass under these conditions. However, the lack of significant changes to BAT marker expression suggests that defective nonshivering thermogenesis may not be the main or sole cause of impaired cold tolerance, yet our analysis suggests that reduced substrate availability due to impaired lipolysis rates could be a partial explanation. The concept of catecholamine resistance has also recently been introduced in obesity and could also contribute to impaired lipid metabolism in our model (Reilly & Saltiel, 2017). While short‐term adrenergic stimulation only revealed defective HSL activation, prolonged cold exposure may further result in impaired lipid metabolism due to altered lipid transport mechanisms. It is therefore important to consider other mechanisms of cold intolerance which could be related to impaired insulation due to loss of AT, yet a poor insulation could lead to an increase in metabolic rates which we did not observe in knockout animals (Cannon & Nedergaard, 2011). Periostin plays a vital role in skin health and its ablation could translate into increased heat loss due to a skin defect that only becomes apparent under challenging conditions (Murota, Lingli, & Katayama, 2017). In summary, the impaired thermoregulation in periostin‐deficient mice is likely a multifactorial process that is accompanied by impaired AT lipolysis and lipid metabolism gene expression during cold. A similar process may also partially contribute to an age‐related defect in AT lipid metabolism (Benjamin, Gellhorn, Wagner, & Kundel, 1961; Gellhorn & Benjamin, 1965).

Chronic overfeeding leads to AT expansion due to adipocyte hypertrophy and is accompanied by enhanced vascularization to assure adequate oxygen supply which requires substantial ECM remodeling (Lemoine et al., 2012). In our model, deletion of *Postn* resulted in a reduced ability for AT expansion during HFD feeding. Given the expression of periostin in cells of the blood vessels, deletion of *Postn* may affect nutrient supply during AT expansion as permeability of the endothelium of blood vessels is pivotal in the release of nutrients to the tissue. Moreover, the substantial reductions in adipose tissue mass in the apparently opposing interventions of cold exposure and HFD suggest that the deletion of *Postn* confers a defect in AT function that is mainly characterized by impaired control of adipocyte size and could be a sign of defective lipid handling. For instance, it was previously shown that changes in adipocyte function due to altered cell size are mediated by activation of integrin/ERK signaling (Farnier et al., 2003). Similarly, genetic ablation of laminin α4, a component of the ECM directly surrounding adipocytes, results in reduced ability to expand in response to diet‐induced and age‐related obesity (Vaicik et al., 2014). This is further supported by the observation of attenuated lipolysis following pharmacological activation of adrenergic signaling, where activation of HSL was impaired in *Postn*‐deficient mice. It remains to be clarified to which extent other tissues are involved in the lipid metabolism phenotype of this animal model with whole‐body deletion of periostin. Aging only leads to a relative reduction in periostin levels that cannot be fully recapitulated in the knockout strain, which persists throughout life and thus would exert phenotypical changes not completely reconstructing the progressive decline of *Postn* expression during aging. Given that periostin expression may also differ between adipose depots and other organs, subsequent studies will require tissue‐specific ablation strategies.

Our study showed that expression of periostin mRNA in human adipose tissue was inversely correlated with BMI but displayed an unexpected positive correlation with aging. This observation could to some extent be explained by interaction with BMI and other parameters of metabolic health as covariates, suggesting that the regulation of periostin gene expression in human WAT responds to different metabolic cues including aging. The discrepant effects of age on periostin gene expression in mice and human white fat remain to be further elucidated but our observations suggest a potential relevance in adipose tissue function. High‐fat feeding in mice induced *Postn* mRNA after a relatively short time course of 6 weeks, while the majority of our study participants had chronically established metabolic dysfunction including insulin resistance or type 2 diabetes, which could help explain this apparent discrepancy. In livers, *Postn* expression is elevated in obese mice and humans, and overexpression of periostin in mouse livers promoted hepatic steatosis and hypertriglyceridemia (Lu et al., 2014). These counteracting processes in liver and AT may explain the limited effects on plasma lipids and glucose metabolism and imply a tissue‐specific regulation and function of periostin. Interestingly, increased circulating periostin was described as a biomarker of increased risk to develop nonalcoholic fatty liver disease and insulin resistance during obesity (Yang et al., 2016). The respective contribution of different tissues to circulating periostin and their involvement in the clinical manifestation of metabolic diseases remains to be clarified in further detail. In our animal studies, periostin expression was induced during HFD feeding. Consequently, the predictive value of periostin as a biomarker may also depend on acute challenges such as nutrition and lifestyle. In summary, these analyses reveal a potential involvement of periostin in metabolic dysfunction of adipose tissue, a novel crosstalk mechanism between the extracellular matrix and adipose tissue lipid metabolism which relays metabolic signals via periostin. Thus, age‐associated metabolic disorders may contribute to defective lipid metabolism by progressive impairment of the extracellular matrix.

## 4 EXPERIMENTAL PROCEDURES

### 4.1 Animal studies

C57BL/6 J mice and *Postn*‐deficient mice (B6 *N*(Cg)‐Postntm1.1(KOMP)Vlcg/J, strain ID: 024,186) were initially purchased from The Jackson Laboratory. Animals were maintained in 12 hr day/night cycles at 20°C (±2°C), with ad libitum access to water and food (Ssniff, Soest, Germany). Except when otherwise stated, young animals between the ages of 10–14 weeks were used for the intervention studies. For HFD, animals were fed a diet containing 45% of calories from fat (Research Diets, New Brunswick, NJ, USA) for 6 weeks starting at 8 weeks of age. Body compositions were assessed using nuclear magnetic resonance (NMR) technology (EchoMRI™‐100H, EchoMRI LLC, Houston, TX, USA). For cold exposure, animals were kept in a climate chamber (Vötsch, Balingen, Germany) at 4°C for 72 hr for males and 24 hr for females. Body temperature of mice was measured using a rectal probe thermometer. For acute β3‐adrenergic stimulation, mice were fasted for 2 hr and were then i.p.‐injected with 1 mg/kg body weight CL316,243 (Sigma‐Aldrich, Taufkirchen, Germany) in PBS. Mice were sacrificed 180 min after injection. A blood sample was collected from the vena facialis. Energy expenditure was measured using indirect calorimetry (PhenoMaster, TSE Systems, Bad Homburg, Germany). Plasma parameters were measured using Cobas Mira technology (Roche Diagnostics, Mannheim, Germany). Plasma insulin was measured using ELISA kits (Crystal Chem, Zaandam, Netherlands). All experimental procedures were approved by the ethics committee for animal welfare and care of the State Office of Environment, Health, and Consumer Protection (State of Brandenburg, Germany).

### 4.2 Isolation of adipogenic progenitors and mRNA analysis

AT‐derived progenitor cells were isolated as described (Schulz et al., 2011; Steinbring, Graja, Jank, & Schulz, 2017). A detailed description of the procedures is supplied in the Supporting Information.

### 4.3 Microarray

BAT‐ and iWAT‐derived APCs of mice of different age groups, that is, 6 and 65 weeks, were sorted by FACS (BD FACSAria™ III, BD Biosciences, San Jose, CA, USA) and cell pellets were immediately lysed in 500 µl TRIzol (Thermo Fisher Scientific) and stored at −80°C before purification using a standard phenol‐chloroform extraction protocol using the RNAqueous Micro Kit (Thermo Fisher Scientific). The transcriptome was analyzed by Microarray using Affymetrix Mouse Exon 1.0 ST Array (Thermo Fisher Scientific) and normalized based upon quantiles. For analysis, samples of three to six animals were pooled per chip and four chips per age group were analyzed. For the pathway analysis, differentially expressed genes with a p‐value below 0.05 were analyzed by DAVID (v. 6.7) software (Huang, Sherman, & Lempicki, 2009, 2009).

### 4.4 Protein analysis

For protein isolation, standard procedures as described in the Supporting Information were used. For quantifications, ImageJ v1.49t (NIH, Bethesda, MA, USA) was used (Schneider, Rasband, & Eliceiri, 2012).

### 4.5 Histological analysis

Tissues were dissected and fixed overnight in 4% formalin at room temperature, dehydrated, and embedded in paraffin. Paraffin sections (4 µm) were stained with hematoxylin and eosin (H&E) or Sirius Red F 3B dye. For measurements of lipid droplet and adipocyte sizes, microscopic images were photographed at either 100× magnification for WAT or 400× magnification for BAT. Adipocyte size was determined using the Wimasis WimAdipose Image Analysis system (Wimasis, München, Germany) and lipid droplet size was measured using ImageJ v1.49t (NIH, Bethesda, MA, USA) (Schneider et al., 2012) as previously described (Parlee, Lentz, Mori, & MacDougald, 2014). A total of three nonoverlapping histological images per animal and three animals per genotype were analyzed. For X‐gal staining, tissues were dissected and fixed in 4% formalin for 1 hr at 4°C. Tissues were rinsed four times for 30 min in rinse buffer (1 × PBS, 2 mM MgCl_2,_0.02% Nonidet P‐40 (NP‐40),0.01% Na‐deoxycholate) and were incubated overnight for β‐galactosidase conversion of X‐gal in a reaction buffer (2 mM MgCl_2,_ 0.02% Nonidet P‐40 (NP‐40), 0.01% Na‐deoxycholate, 5 mM potassium ferrocyanide, 5 mM potassium ferrocyanide, 1 mg/ml X‐gal) at 37°C in the dark. Subsequently, tissues were washed in PBS (four times for 20 min), dehydrated and embedded in paraffin. Sections were counterstained with H&E.

### 4.6 Human studies

Our study includes data from 471 participants, who have been recruited at the University Hospital in Leipzig (Germany) as previously described (Guiu‐Jurado et al., 2016). All studies were approved by the ethics committee of the University of Leipzig (approval numbers: 159–12–21,052,012 and 017–12–23,012,012) and all subjects gave written informed consent before taking part in the study. Paired samples of abdominal omental, visceral, and subcutaneous adipose tissue were obtained from 471 Caucasian men (*n* = 146) and women (*n* = 325), who underwent open abdominal surgery as described previously (Guiu‐Jurado et al., 2016). The age ranged from 19 to 93 years and BMI from 19 to 76 kg/m^2^. Adipose tissue was immediately frozen in liquid nitrogen and stored at −80°C. RNA was extracted from adipose tissue using RNeasy Lipid tissue Mini Kit (Qiagen, Hilden, Germany). Quantity and integrity of RNA was monitored with NanoVue plus Spectrophotometer (GE Healthcare, Freiburg, Germany). One microgram total RNA from sWAT and vWAT was reverse‐transcribed with standard reagents (Life Technologies). cDNA was then processed for TaqMan probe‐based quantitative RT–PCR using the QuantStudio 6 Flex Real‐Time PCR System (Life Technologies). Human periostin expression was measured by quantitative real‐time RT–PCR using the following probes: human periostin (Hs01566750_m1). Fluorescence emissions were monitored after each cycle. Human periostin mRNA expression was calculated relative to the mRNA expression of *hypoxanthine guanine phosphoribosyltransferase 1 (HPRT1)* (Hs01003267_m1). Correlations were evaluated using Spearman partial correlation analyses adjusted indicated parameters. Due to non‐normal distribution of the gene expression variables, Spearman rather than Pearson correlation analysis was employed. Fisher's *z* transformation was used to produce 95% confidence intervals for each correlation coefficient. All statistical analyses were performed using SAS (version 9.4, Enterprise Guide 6.1, SAS Institute Inc., Cary, NC, USA).

### 4.7 Statistical analysis

Statistical differences between groups were evaluated using either an unpaired two‐tailed Student's *t* test, Mann–Whitney U test, or analysis of variance (ANOVA) with Bonferroni *post hoc* test.

## CONFLICT OF INTEREST

The authors declare no conflict of interests.

## AUTHORS’ CONTRIBUTION

T.J.S and A.G. designed the study, analyzed the data, and wrote the manuscript. A.G performed the majority of the experiments. A.M.J, S.G., F.G.C., W.J., and T.H.A. performed some experiments and critically reviewed the manuscript. S.U. and A.S. provided valuable research materials and reviewed the manuscript. M.B. and M.K. provided the human data and helped analyzing the data. K.A. performed the correlation analyses for the human data.
