# Supplementary material for: Loss of periostin occurs in aging adipose tissue of mice and its genetic ablation impairs adipose tissue lipid metabolism
Source: Aging Cell. 2018 Aug 7;17(5):e12810. doi: 10.1111/acel.12810 (PMC6156450; doi:10.1111/acel.12810)
Supplement: Supplementary file 1 [file ACEL-17-e12810-s001.pdf]

Graja et al. Supplementary Figure 1

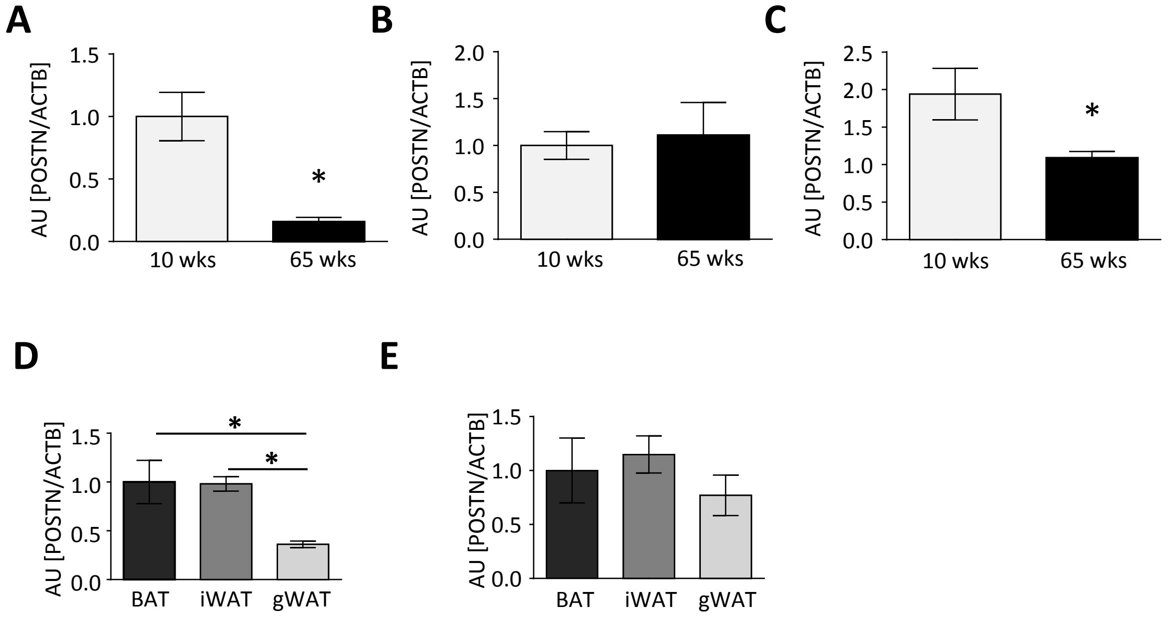

Graja et al. Supplementary Figure 2

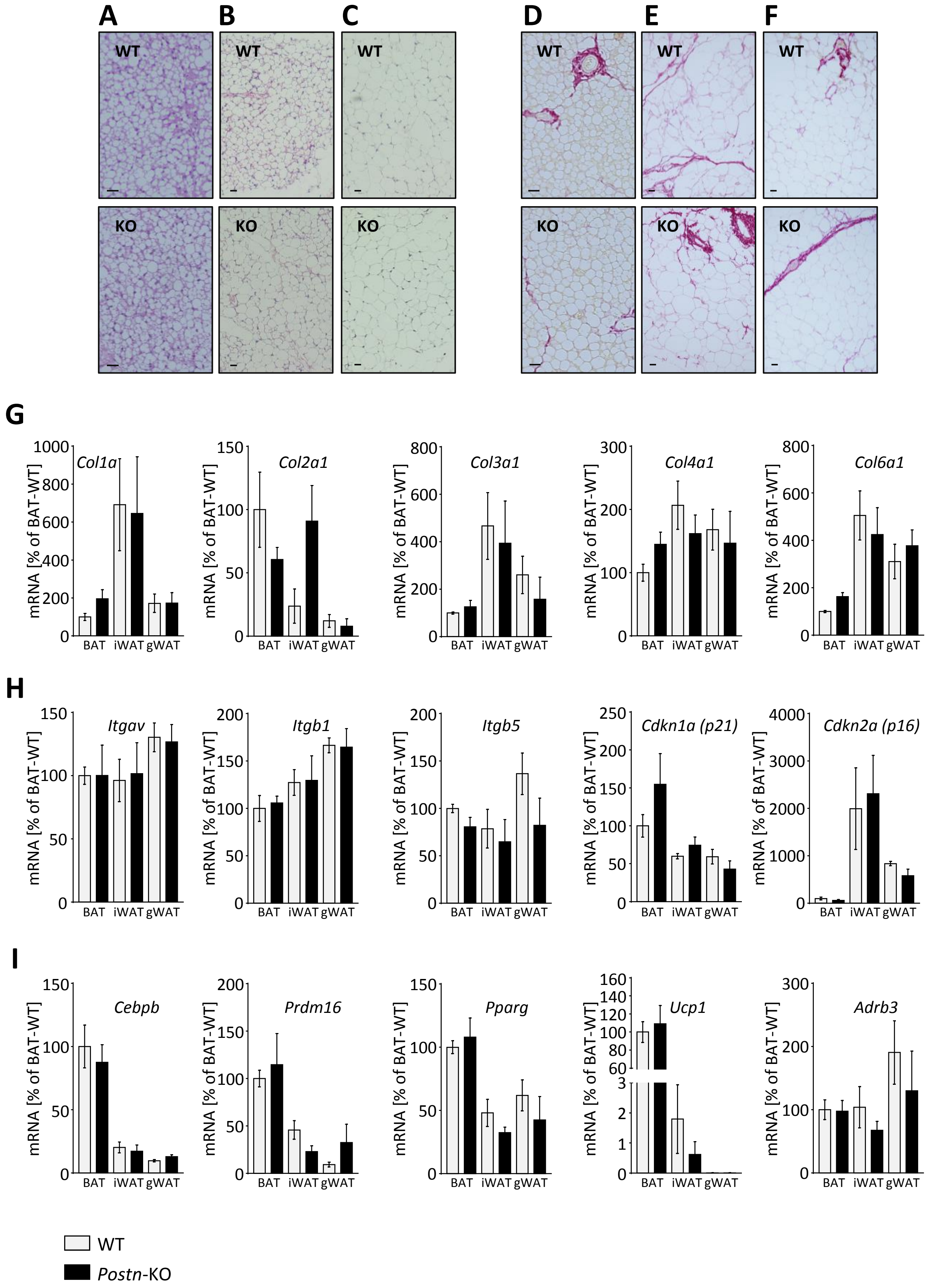

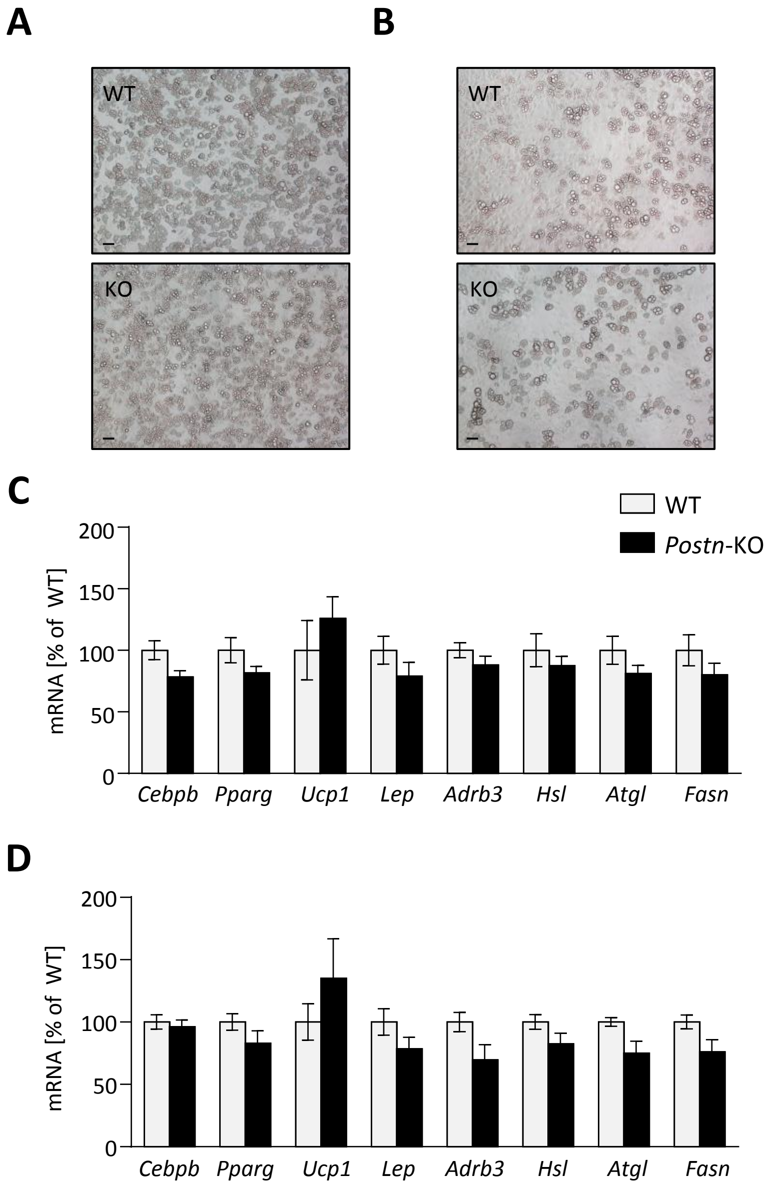
A
B
WT
KO
WT
KO
C
WT
Postn-KO
mRNA [% of WT]
200
150
100
50
0
Cebpb
Pparg
Ucp1
Lep
Adrb3
Hsl
Atgl
Fasn
D
mRNA [% of WT]
200
150
100
50
0
Cebpb
Pparg
Ucp1
Lep
Adrb3
Hsl
Atgl
Fasn

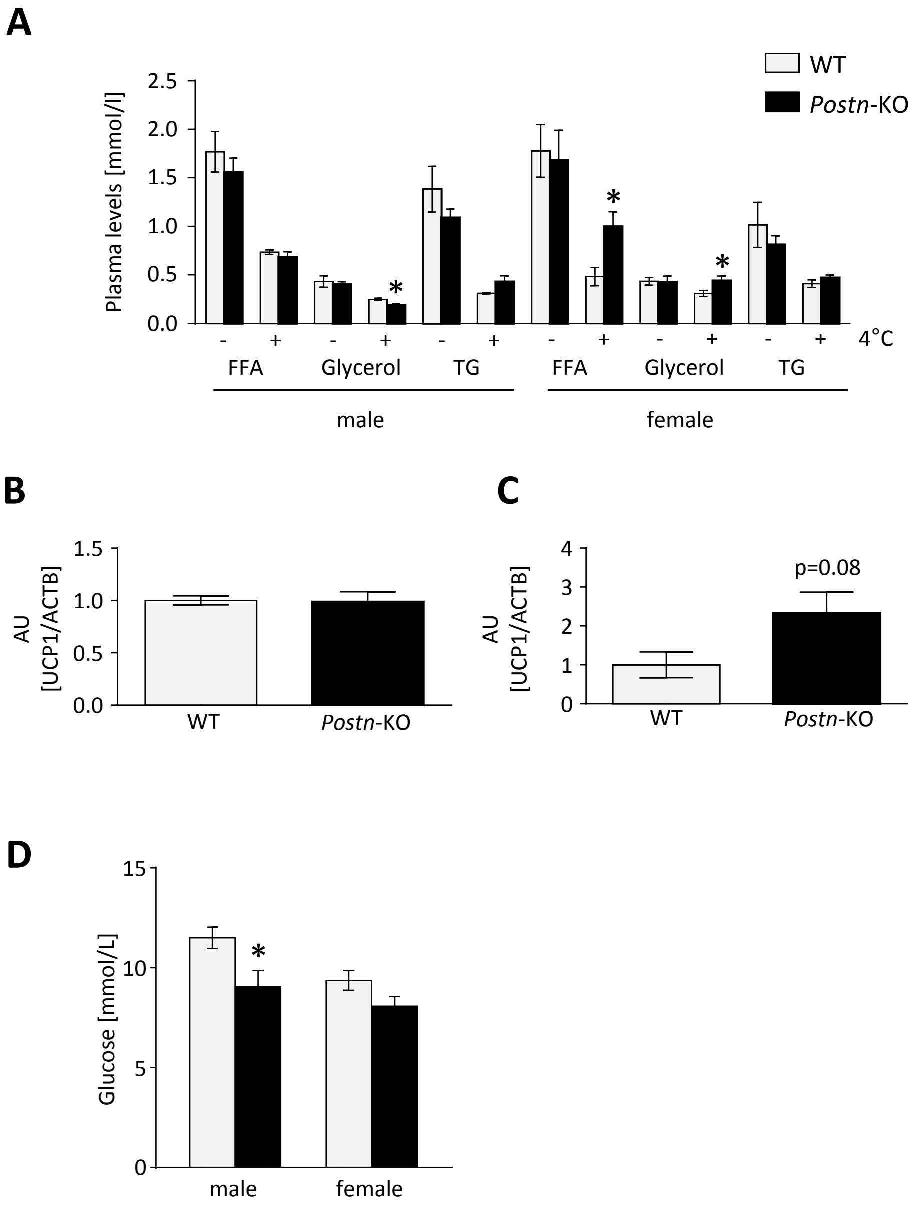
A
WT
Postn-KO
Plasma levels [mmol/l]
2.5
2.0
1.5
1.0
0.5
0.0
4°C
FFA
Glycerol
TG
male
female
B
AU
[UCP1/ACTB]
1.5
1.0
0.5
0.0
WT
Postn-KO
C
AU
[UCP1/ACTB]
p=0.08
4
3
2
1
0
WT
Postn-KO
D
Glucose [mmol/L]
15
10
5
0
male
female

Graja et al. Supplementary Figure 5

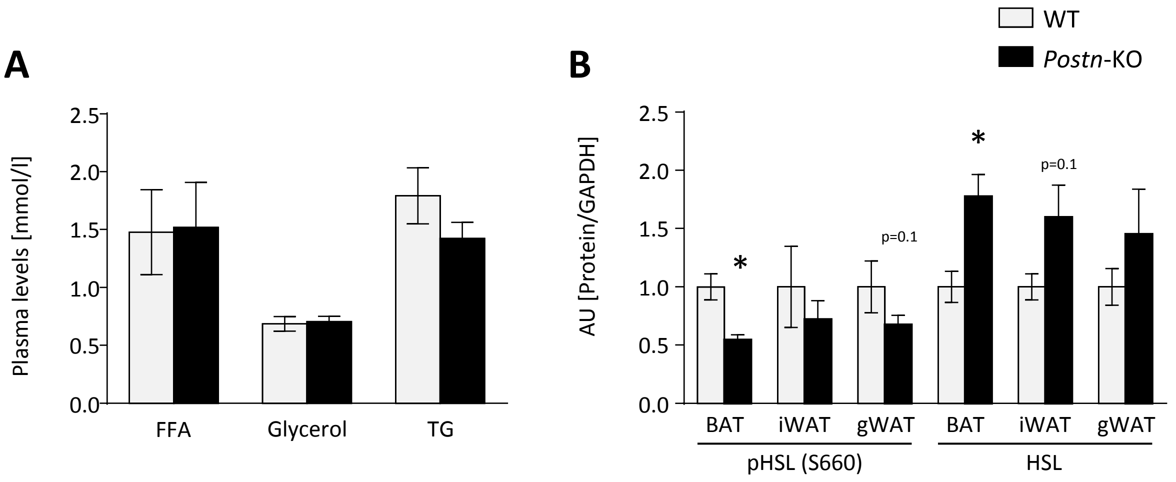

Graja et al. Supplementary Figure 6

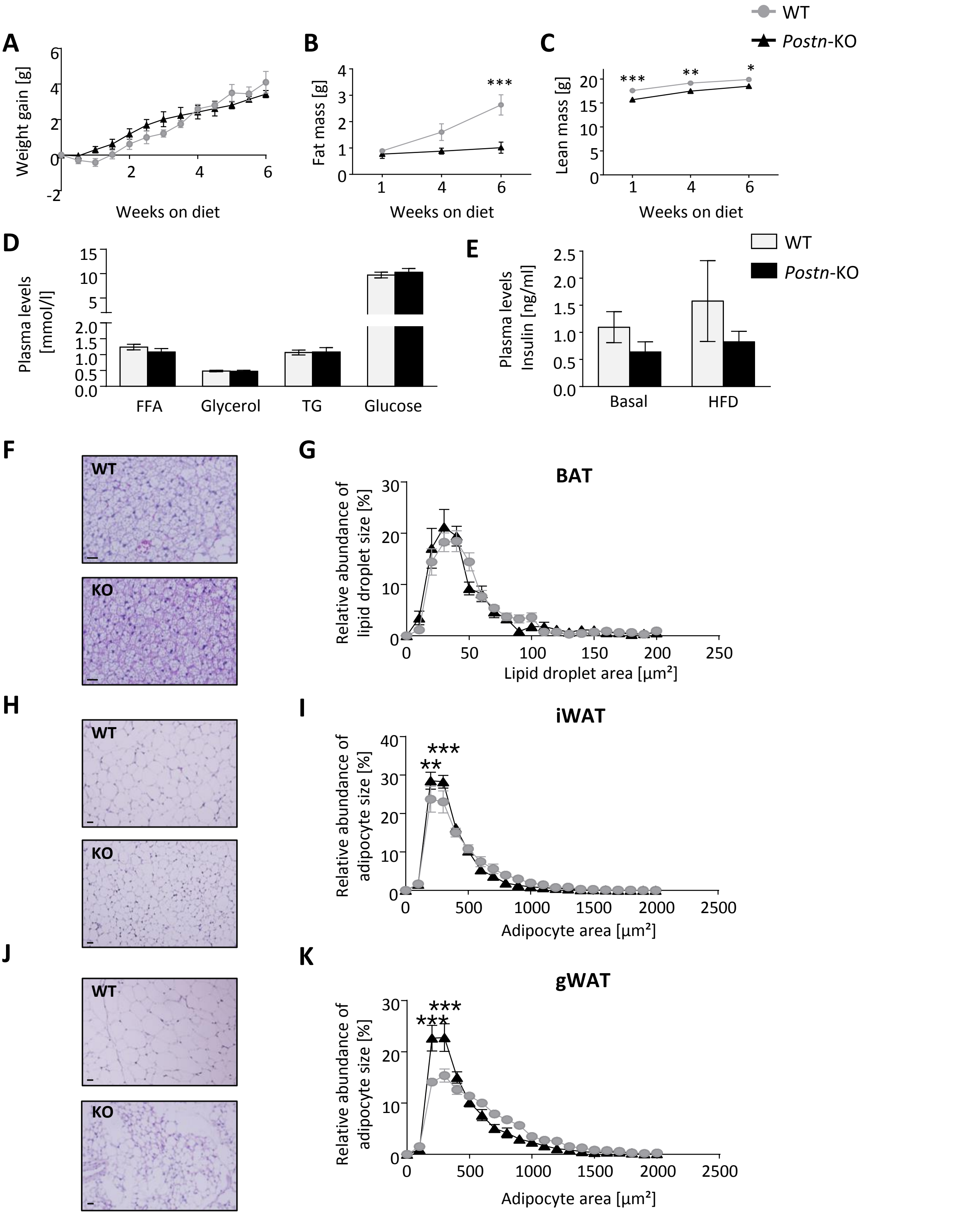

Graja et al. Supplementary Figure 7

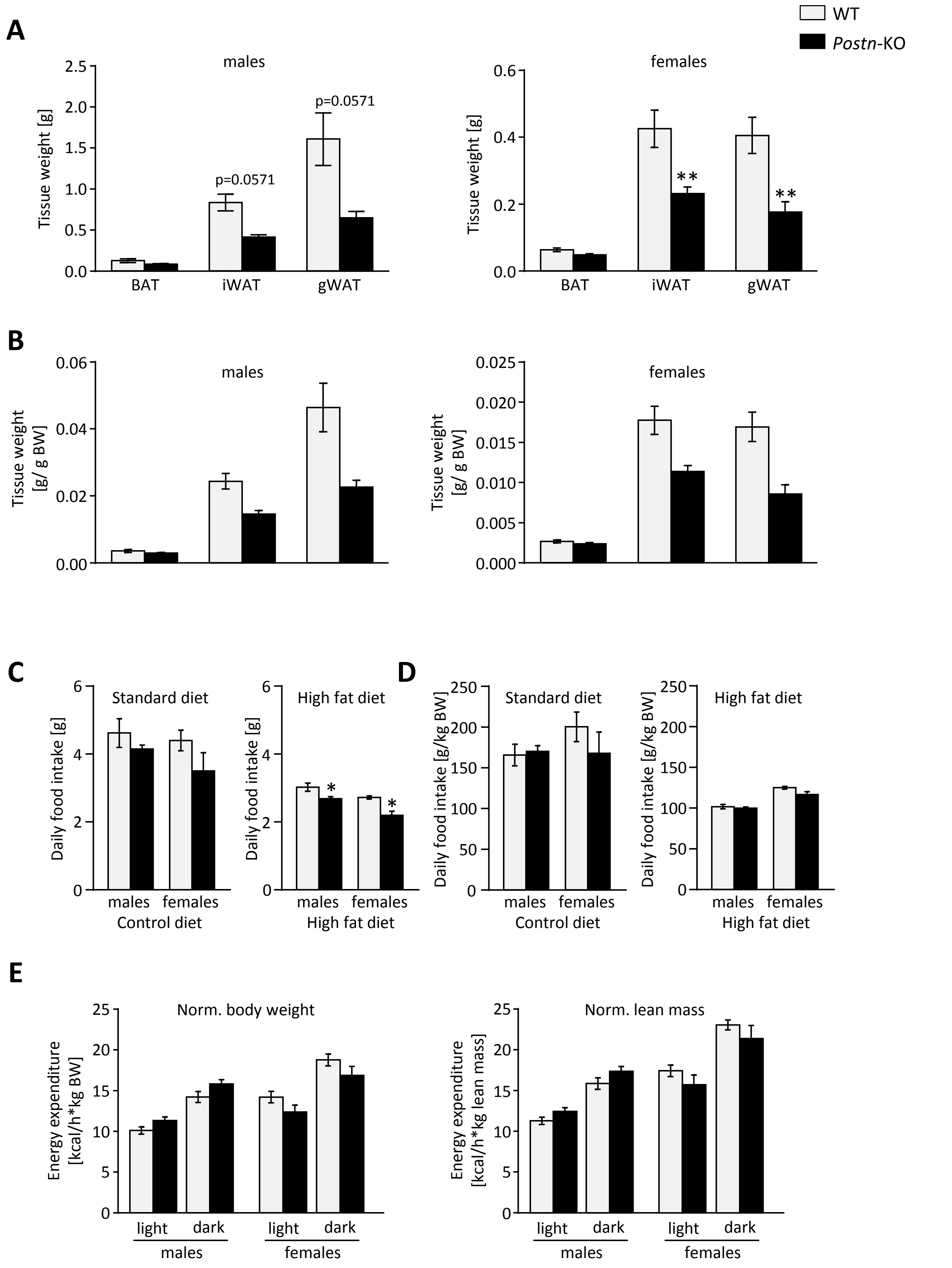

Graja et al. Supplementary Figure 8

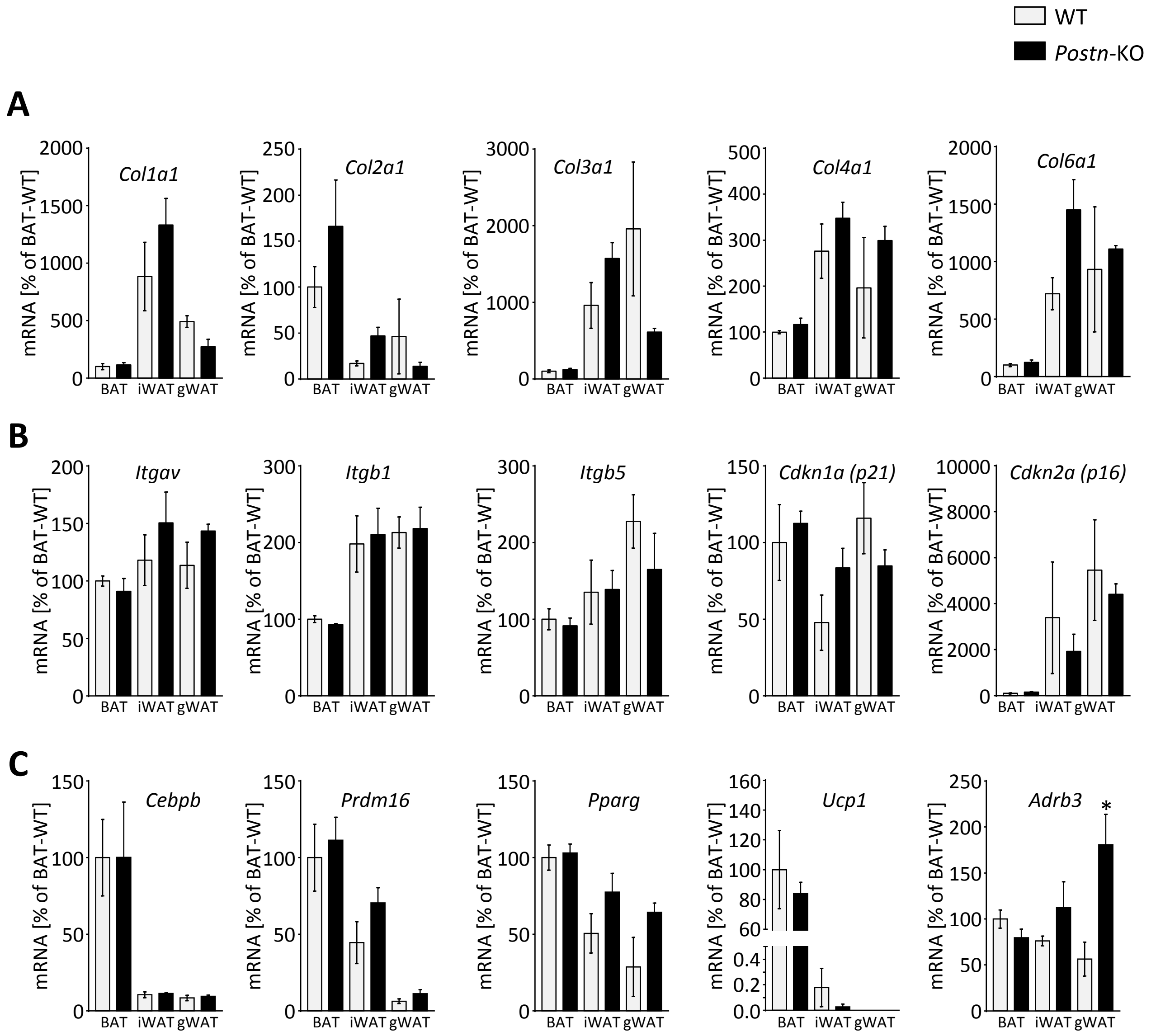

Supplementary Figure 1

(**A**-**C**) Quantification of POSTN protein expression by western blot (as shown in main Figure item 1D) normalized to β-Actin (ACTB) in iWAT (A) and gWAT (B), and BAT (C) of young (10 weeks) compared to old (65 weeks) mice.

(**D**, **E**) Comparative POSTN protein quantification by western blot (as shown in main Figure items 1H and 1I) normalized to ACTB in BAT, iWAT and gWAT of male (H) and female (I) mice. Data are shown as mean ± SEM. n=5-7. *$p<0.05$ as assessed by Mann Whitney U test.

Supplementary Figure 2

(**A**) Representative images of H&E staining (200x magnification; Scale bar: 20 µm) of BAT of male WT (upper panel) and male *Postn*-KO mice (lower panel) maintained on basal/ control diet.

(**B**, **C**) Representative images of H&E staining (100x magnification; Scale bar: 20 µm) of iWAT (B) and gWAT (C) of male WT (upper panel) and male *Postn*-KO mice (lower panel) maintained on basal/ control diet.

(**D**) Representative images of Sirius red staining (200x magnification; Scale bar: 20 µm) of BAT of male WT (upper panel) and male *Postn*-KO mice (lower panel) maintained on basal/ control diet.

(**E**, **F**) Representative images of Sirius red staining (100x magnification; Scale bar: 20 µm) of iWAT (E) and gWAT (F) of male WT (upper panel) and male *Postn*-KO mice (lower panel) maintained on basal/ control diet.

(**G**-**I**) mRNA levels of ECM genes (G: Col1a, Col2a1, Col3a1, Col4a1, Col6a1), integrins (H: Itgav, Itgb1, Itgb5), senescence markers (H: p21, p16) and brown adipogenic markers (I: Cebpb, Prdm16, Pparg, Ucp1, Adrb3) in BAT, iWAT, and gWAT of wildtype control (gray bars) and *Postn* knockout mice (black bars) maintained on basal/ control diet. Data are shown as mean ± SEM.

Supplementary Figure 3

(**A**, **B**) Representative microscopic images (100x magnification; scale bar: 50 µm) of differentiated primary adipogenic progenitor cells derived either from WT mice (upper panel) or *Postn*-KO mice (lower panel). Cells were isolated either from BAT (A) or iWAT (B).

(**C**, **D**) Gene expression of differentiated primary adipogenic progenitor cells derived from BAT (C) and iWAT (D) of WT and *Postn*-KO mice. Data are shown as mean ± SEM (n=3).

Supplementary Figure 4

(**A**) Plasma levels of free fatty acids (FFA), glycerol and triacylglycerides (TG) in male and female WT and *Postn*-KO animals before and after cold exposure for 72 h (males) and 24 h (females; n=5-7).

(**B**, **C**) Comparative UCP1 protein quantification by western blot (as shown in main Figure items 3F and 3G) normalized to β-Actin (ACTB) in BAT (B) and iWAT (C) of male mice after cold exposure (n=4-7).

(**D**) Plasma glucose levels in male and female WT and *Postn*-KO animals before and after cold exposure for 72 h (males) and 24 h (females; n=5-7).

Data are shown as mean ± SEM. *p<0.05 compared with WT animals assessed by unpaired t-test or Mann Whitney U test.

Supplementary Figure 5

(**A**) Plasma free fatty acid, glycerol, and triglyceride levels in animals after 180 min Cl316,243 treatment.

(**B**) Western blot quantification of pHSL and HSL in BAT, iWAT, and gWAT (as shown in main Figure item 4C). Western blot signals were normalized to Glyceraldehyde 3-phosphate dehydrogenase (GAPDH).

Data are shown as mean ± SEM. *p<0.05 compared with WT animals assessed by Mann Whitney U test.

Supplementary Figure 6

(**A**) Body weight gain of female WT and *Postn*-KO animals during 6 weeks of HFD feeding. Gray lines/circles/bars represent WT controls, black lines/triangles/bars represent *Postn*-KO mice, applies to all subsequent panels.

(**B**, **C**) NMR analysis of female WT and *Postn*-KO animals assessing total fat mass (B) and lean mass (C).

(**D**) Plasma levels of FFA, glycerol, TG, and glucose in female WT and *Postn*-depleted animals after 6 weeks of HFD.

(**E**) Plasma levels of insulin with and without high-fat diet feeding in female WT and *Postn*-depleted animals.

(**F**) Representative images of H&E staining (200x magnification; Scale bar: 20 µm, applies to all subsequent images of BAT) of BAT of female WT (upper panel) and female *Postn*-KO mice (lower panel).

(**G**) Quantitative analysis of lipid droplet size in BAT sections after H&E staining of female WT and knockout animals after 6 weeks of HFD as shown in previous panel.

(**H**) Representative H&E staining (100x magnification; Scale bar: 20 µm, applies to all subsequent images of WAT) of iWAT of female WT (upper panel) and female *Postn*-KO mice (lower panel).

(**I**) Adipocyte size analysis of female iWAT comparing WT to *Postn*-KO animals after 6 weeks of HFD from images as shown in previous panel.

(**J**) Representative H&E staining (100x magnification) of gWAT of female WT (upper panel) and female *Postn*-KO mice (lower panel).

(**K**) Adipocyte size analysis of female gWAT comparing WT to *Postn*-KO animals after 6 weeks of HFD from images as shown in previous panel.

Data are shown as mean ± SEM (n=5-7). *$p<0.05$, **$p<0.01$, ***$p<0.001$ as assessed by two-way ANOVA with Bonferroni *post hoc* test (A, B, G, I, K) and unpaired t-test (D, E).

Supplementary Figure 7

(**A**) Adipose tissue weights in male (left panel) and female (right panel) WT and *Postn*-KO mice after 6 weeks of HFD (n=3-6). Grey bars depict wildtype control animals, black bars depict *Postn*-KO animals (applies to all subsequent panels).

(**B**) Adipose tissue weights in male (left panel) and female (right panel) WT and *Postn*-KO mice after 6 weeks of HFD after normalization to body weight (n=3-6).

(**C, D**) Average daily food intakes of male and female WT and *Postn*-KO mice fed standard (C) or high fat diet (D) before (left panels) and after (right panels) normalization to body weight (n=5-7).

(**E**) Energy expenditure in animals fed control diet during light (6 am-6 pm) and dark (6 pm-6 am) phases after normalization to body weight or lean mass (n=5-7).

Data are shown as mean ± SEM. *p<0.05, **p<0.01as assessed by Mann-Whitney test (A,B,D) or ANOVA (C).

Supplementary Figure 8

(**A**-**C**) mRNA levels of ECM genes (A: *Col1a, Col2a1, Col3a1, Col4a1, Col6a1*), integrins (B: *Itgav, Itgb1, Itgb5*), senescence markers (B: *p21, p16*) and brown adipogenic markers (C: *Cebpb, Prdm16, Pparg, Ucp1, Adrb3*) in BAT, iWAT, and gWAT of wildtype control (gray bars) and *Postn* knockout mice (black bars) maintained on HFD for 6 weeks. Data are shown as mean ± SEM. *p<0.05, as assessed by Mann-Whitney test.

Supplementary Table 1. Spearman correlation coefficients (Rhos) and 95% confidence intervals (CI-s) for depicting associations between participants' age and POSTN mRNA expression in sWAT and vWAT according to categories of body mass index (BMI), 95% CI calculated using Fisher's z transformation.

	BMI<25 kg/m^2 (n=32)		25<BMI≤30kg/m^2 (n=28)		BMI≥30 kg/m^2 (n=408)	
	R_{ho} (95% CI)	*P*-value	R_{ho} (95% CI)	*P*-value	R_{ho} (95% CI)	*P*-value
POSTN mRNA Human sWAT[AU]	-0.15 (-0.47; 0.20)	0.39	0.22 (-0.16; 0.54)	0.25	-0.03 (-0.06; 0.13)	0.47
POSTN mRNA Human vWAT[AU]	-0.19 (-0.50; 0.16)	0.28	0.27 (-0.12; 0.58)	0.16	0.08 (0.00;0.18)	0.08

Supplementary Table 2. Intron-spanning primers used for mRNA quantification by real-time RT-PCR.

Gene	Sequence (5'→3')
Adrb3	Fwd: GGCCCTCTCTAGTTCCCAG<br>Rev: TAGCCATCAAACCTGTTGAGC
Atgl	Fwd: TGACCATCTGCCTTCCAGA<br>Rev: TGTAGGTGGCGCAAGACA
Cebpb	Fwd: CAAGTTCCGCAGGGTGCT<br>Rev: CCAAGAAGACGGTGGACAA
Ces1d	Fwd: TGGTATTTGGTGTCCCATCA<br>Rev: GCTTGGGCGATACTCAAACT
Fasn	Fwd: CTCGCTTGTCGTCTGCCT<br>Rev: TTGGCCCAGAACTCCTGTAG
Hsl	Fwd: ACGCTACACAAAGGCTGCTT<br>Rev: TCTCGTTGCGTTTGTAGTGC
Postn (mouse)	Fwd: CGGGAAGAACGAATCATTACA

	Rev: ACCTTGGAGACCTCTTTTTGC
Pgc1a	Fwd: CCCTGCCATTGTTAAGACC<br>Rev: TGCTGCTGTTCCTGTTTTC
Pparg	Fwd: CTCCAAGAATACCAAAGTGCGA<br>Rev: GCCTGATGCTTTATCCCCACA
Ppia	Fwd: CAAATGCTGGACCAAACACAA<br>Rev: AAGACCACATGCTTGCCAT
Prdm16	Fwd: CAGCACGGTGAAGCCATTC<br>Rev: GCGTGCATCCGCTTGTG
Rplp0	Fwd: TTTGGGCATCACCACGAAAA<br>Rev: GGACACCCTCCAGAAAGCGA
Ucp1	Fwd: CAAATCAGCTTTGCCTCACTC<br>Rev: TAAGCCGGCTGAGATCTTGT

Isolation of adipogenic progenitors and mRNA analysis

In brief, cells positive for surface markers Stem cell antigen 1 (Sca1; 1:400; #17-5981-82, Thermo Fisher Scientific, Dreieich, Germany) and negative for the hematopoietic marker CD45 (1:200; #11-0451-82, Thermo Fisher Scientific) and the endothelial marker CD31 (1:100; #11-0311-82, Thermo Fisher Scientific), were purified by fluorescence-activated cell sorting (FACS, BD FACSAria™ III, BD Biosciences, San Jose, CA, USA). Cells were either analyzed by microarray or gene expression analysis or cultivated as described previously (Steinbring *et al*. 2017). For RNA isolation, tissues were dissected and mortar-ground while frozen in liquid nitrogen and subsequently homogenized with a tissue homogenizer (Bullet Blender, Next Advance, Averill Park, NY, USA) in 200 µl of Qiazol (Qiagen, Hilden, Germany) and an equal volume of 0.9-2.0 mm stainless steel beads (Biostep, Burkhardtsdorf, Germany). Lysates were centrifuged for 5 min at 4°C at 2000 rpm and the liquid supernatant underneath the fat layer was collected and the RNA was purified using a commercially available RNA MiniPrep Kit (Zymo Research, Freiburg, Germany). Purified RNA was transcribed into cDNA with the high capacity cDNA reverse transcription kit (Thermo Fisher Scientific). mRNA was quantified using Maxima SYBR Green/ROX qPCR Master Mix (Thermo Fisher Scientific) and the Real-Time PCR system CFX384 Touch (Bio-Rad, München, Germany) and using intron-spanning primers (Suppl. Table 1). mRNA expression was calculated relative to the mRNA expression of Cyclophilin (*Ppia*) or Acidic ribosomal phosphoprotein P0 (*Rplp0)*.

Protein analysis

For protein isolation, tissues were ground while frozen in liquid nitrogen and 200 µl of RIPA buffer with phosphatase inhibitor and proteinase inhibitor were added. Tissue samples were homogenized with a hand held pestle and protein concentrations were determined with a BCA protein assay kit (Thermo Fisher Scientific). The protein samples were mixed with 5x Laemmli buffer, boiled at 95°C for 5 min in presence of 10 % β-mercaptoethanol and separated via SDS-polyacrylamid gel electrophoresis and transferred to polyvinylidene difluoride membrane. Proteins were detected using the following

antibodies: anti-UCP1-ab (1:1000, Genetex, Irvine, CA, USA), anti-POSTN-ab (1:1000, Life Technologies, Darmstadt, Germany), anti-ATGL-ab (1:1000, Cell signaling Technology, Danvers, MA, USA), anti-CD36-ab (1:1000, Cell signaling Technology, Danvers, MA, USA), anti-HSL-ab (1:1000, Cell signaling Technology, Danvers, MA, USA), anti-Perilipin1-ab (1:1000, Progen Biotechnik GmbH, Heidelberg, Germany), anti-phospho (S660) HSL-ab (1:1000, Cell signaling Technology, Danvers, MA, USA), followed by incubation with a horseradish-peroxidase conjugated secondary antibody. Proteins were visualized using ECL detection (Thermo Fisher Scientific) and a chemiluminescence imager (Peqlab Biotechnology, Erlangen, Germany). As a loading control, a total protein stain, anti-β-Actin HRP-conjugated (1:10,000, Sigma Aldrich) and anti-Glyceraldehyde-3-phosphate dehydrogenase HRP-conjugated (1:1000, Cell signaling Technology, Danvers, MA, USA) were used.
